# Emotions during the Covid‐19 pandemic: Fear, anxiety, and anger as mediators between threats and policy support and political actions

**DOI:** 10.1111/jasp.12806

**Published:** 2021-06-17

**Authors:** Emma A. Renström, Hanna Bäck

**Affiliations:** ^1^ Department of Psychology University of Gothenburg Gothenburg Sweden; ^2^ Department of Political Science Lund University Lund Sweden

## Abstract

The Covid‐19 pandemic has significantly changed the lives of most people. It has been described as the most severe global health disaster of modern times by the United Nations. No doubt such a major crisis influences what citizens think of different policies, and how they become politically active, not to mention, the forceful emotional experiences that the Covid‐19 pandemic brings. This study evaluates how emotions affect support for policies related to restricting the spread of the virus and economic assistance, and how emotions affect intentions to engage politically. In an experiment (*N* = 1,072), we manipulated emotional reactions to threat by highlighting different aspects of the pandemic. Our findings show that different experimental treatments elicit different emotions, and that fear, anxiety, and anger are all related to policy support and political action intentions, but in different ways. Fear and anger predict support for restrictive policies to limit the spread of the virus, while anxiety predicts support for economic policies. Anger and anxiety, but not fear, increase intentions to engage politically. Hence, we find support for a mechanism where different aspects of the Covid‐19 crisis evoke different emotional reactions, which in turn affects policy support and political actions differently.

## 1 INTRODUCTION

The Covid‐19 pandemic has shaken the world in its foundations and has had a significant impact on individual and public health (e.g., Arora & Grey, 2020). If we are to understand how health behaviors are affected by the pandemic, we also need to understand what kind of policies citizens endorse. The governmental reactions to the pandemic—how to limit the spread of the virus while at the same time keep a society alive, differed widely between countries. In the initial phase, the most common strategy in the Western European countries was complete lockdowns with the main goal to stop the virus from spreading. That is, social confinement was in most countries nationwide and strictly enforced (Bol et al., 2020). Countries such as Austria, France, Norway, Italy, UK, Denmark, and the Netherlands had all adopted lockdown policies by the end of March 2020.

Two major problems with this approach have been noted. First, it is impossible to keep a complete lockdown for an extended period of time—both for economic and health reasons (Holmes et al., 2020; Huang & Zhao, 2020; Li et al., 2020). Second, even though lockdowns successfully reduced the spread of the virus, a second wave was feared when restrictions were lifted. As it later turned out, this fear was warranted since lifting the restrictions led to an increase in spread and many countries have to date (March 2021), enforced several lockdown periods.

The public's reactions to the different strategies also varied extensively. In times of crisis, support for the government and its actions often increase (Campbell, 2012), which was also the case in the initial phase of Covid‐19 (Bol et al., 2020). However, as time went by and the confinement of lockdowns exerted psychological and economic stress, many became more negative. In fact, several countries have even faced civil unrest. It is clear that this pandemic, and the governmental responses to it, has brought with it a host of emotions, ranging from anger to fear and anxiety. In this article, we explore how emotions function as mediators, explaining the effect of the saliency of different aspects of the Covid‐19 pandemic, on support for different policies and action intentions.

## 2 EMOTIONS IN POLITICAL PSYCHOLOGY

In clinical and health psychology, there is a long tradition of taking emotions into account. Even though this perspective has been less present in political psychology, a growing literature explores how affect influences political actions and attitudes (Brader & Marcus, 2013; Houghton, 2009; Lambert et al., 2019). Affect is a broad term that includes both emotions and mood. While the mood is generally not associated with a particular stimulus, emotions are described as “elicited by something, are reactions to something, and are generally about something” (Ekkekakis, 2013, p. 322). In addition, emotions tend to be fairly quick responses that also fade fairly quickly. In the present research, we are primarily interested in emotions as reactions to specific information related to the Covid‐19 pandemic.

Emotions are important to human life in general, and thus also to political life. Emotions inform individuals about a situation and prepare the body for a certain course of action (Frijda, 1986). Emotions also affect cognitive processing such as attention, information seeking, and reliance on heuristics and stereotypes (Brader & Marcus, 2013). As such, emotions have an important place in explaining political behavior and attitudes. For instance, it is well‐established that emotions are important predictors in collective action and political activism (Goodwin et al., 2001; Gould, 2009; Klandermans & Stekelenburg, 2013), as well as influencing how political information is processed and how political decisions are made (Brader, 2006), and political attitudes in a broader sense (Brader & Marcus, 2013; Lambert et al., 2019).

There are several different theories on how emotions should be conceived of, ranging from a set‐up of distinct emotions, to a dimensional approach where different emotional states are more fluid. Regardless, a broad distinction is made by focusing on valence, that is, the positive or negative nature of emotions. In the past, negative emotions have often been lumped together, regardless of their specific nature, including anger, fear, anxiety, and sadness (see, e.g., Brader & Marcus, 2013 for an overview). The widely influential PANAS is an example of this. PANAS stands for Positive Affect (PA) and Negative Affect (NA) and suggests that affect should be conceptualized in terms of these two dimensions (Watson et al., 1988). However, research suggests that different negative emotions have a different impact on cognition and behavior. In contemporary political psychology, one of the most common distinctions is that between fear/anxiety and anger. There is much evidence that fear and anxiety, compared to anger, differently impact cognition and behavior (Banks & Valentino, 2012; Bodenhausen et al., 1994; Marcus et al., 2000; Merolla & Zechmeister, 2009).

### 2.1 The effect of anger on political attitudes and behavior

Anger is one of the most well‐researched emotions in psychology in general, including clinical psychology. Hence, there is much work on its causes and consequences both on cognition and behavior. Anger has been described as a goal‐pursuing and approach‐linked emotion, motivating action, and decreasing cognitive processing. Anger is related to physical activity and increased blood flow (“fight” response). Several studies have shown that anger leads to confrontation and approach (Carver & Harmon‐Jones, 2009; Harmon‐Jones et al., 2009, 2014; Huddy et al., 2007).

The fact that anger has been in the empirical spotlight for such a long time makes anger a good candidate to apply to a political context, and not surprisingly, anger is one of the most explored emotions in political psychology. Anger has been shown to be a strong motivation for participation in collective actions, such as demonstrations (Klandermans & van Stekelenburg, 2013). Anger has a mobilizing function, which was for example shown by Valentino and colleagues (2011) in their influential paper on the mobilizing effect of anger on voting behavior. One reason why anger has this mobilizing function is that anger is elicited by perceived unfairness. Anger is the signal that something must be done to make things just (Huddy et al., 2007; Lambert et al., 2019). Hence, angry people are more likely to engage. Anger has also been related to political attitudes and policy support, even though the literature here is not as extensive. For instance, anger, but not fear or anxiety, has been related to support for “hawkish,” high‐risk governmental policies (Lerner et al., 2003; Sadler et al., 2005; Skitka et al., 2006).

### 2.2 The effect of fear and anxiety on political attitudes and behavior

Less attention has been paid to the difference between fear and anxiety, even though there are some indications that these two emotions could have distinctive effects as well (Sylvers et al., 2011). We here draw on Brader and Marcus (2013), who suggest that a possible distinction between fear and anxiety could be important for future research in the field of political psychology. In the present study, we explore these two emotions as separate and argue that fear and anxiety should have distinctive effects on political attitudes and behavior.

The labels fear and anxiety are often used interchangeably in the political psychology literature (Brader & Marcus, 2013; Wagner & Morisi, 2019). Fear and anxiety share many more features as compared to for instance anger, even though all three are considered negative emotions (Watson et al., 1988). Both fear and anxiety are elicited by threats and serve the evolutionary purpose of survival (Lang et al., 2000). Even though fear and anxiety in the social and political psychology literature most often are treated as reflecting the same underlying emotion, but perhaps of varying intensity, clinical researchers distinguish between anxiety (such as Generalized Anxiety Disorder) and fear (present in specific phobias) (Öhman & Mineka, 2001). In DSM‐5 (The diagnostic and statistical manual of mental disorders, APA, 2013), anxiety is described as a worry about future events, whereas fear is a reaction to current events. Moreover, fear and anxiety seem to operate through different neural pathways and have different defining characteristics (Sylvers Lilienfeld & LaPrairie, 2011).

Fear is a fast emotion in that it is elicited when a threat is imminent or clear and the response also quickly fades. Fear is elicited when a threat is perceived to target the self (Davis & Stephan, 2011). Fear is associated with a sense of danger and motivates fleeing behavior, which also suggests that fear is a high‐arousal emotion, just like anger. Anxiety, however, is elicited when the threat is ambiguous, uncertain, or less specified, and the reaction is a prolonged emotional state. Since the threat is not defined, anxiety does not motivate fleeing in the same manner as fear and has been shown to lead to both approach and avoidance reactions (Gray & McNaughton, 2000; Perkins et al., 2012). Previous research on fear has shown that fear increases risk estimates and precautionary planning (Lerner et al., 2003). Fear also evokes uncertainty and feelings of lack of individual control, which are central in determining risks (Lerner & Keltner, 2001; Slovic, 1987).

There is an extensive body of research in political psychology about how threat affects political attitudes and political behavior. Although, it should be noted that emotions are usually not measured in this line of research (Lambert et al., 2019), which means that even if threat has been found to influence attitudes and behavior, the specific emotion elicited by the threat is not empirically investigated. Much of the previous research that has examined the relationship between threat and political attitudes or behavior have focused on intergroup threat (such as terrorist threat, or the threat of immigration). The observed following attitude shift has also mainly been related to political attitudes relating to the threat, such as more restrictive immigration policies, increased military spending following the 9/11 terrorist attacks, but not on other issues related to ideology such as feminism or socialized medicine (Nail & McGregor, 2009).

Research from the terror management literature has shown that the existential threat elicited by the thought of an unavoidable and imminent death causes existential anxiety (Greenberg et al., 1990; Pyszczynski et al., 2003). Mortality salience has been linked to political conservatism, increased nationalism, and Republican voting. This effect has been labeled the “conservative shift” hypothesis (Arndt et al., 2002; Jost et al., 2003; Landau et al., 2004), and is based on research that has linked existential anxiety with a low tolerance for ambiguity. Relatedly, a recent study found that existential anxiety was related to voting for the more secure, status quo, option in the Brexit referenda (Bäck et al., 2020). Research that focuses on the role of existential threat in the mortality salience literature, has found mixed effects on political attitudes (Burke et al., 2010). A possible explanation for such mixed results is the lack of emotion measures that could function as mediators.

The research mentioned above has mainly used experimentally induced anxiety, but there are also individual variations in trait anxiety. One study investigated the relation between trait anxiety and political attitudes and found weak relations that anxiety disorders were related to concerns about economic inequality and environmental issues (Helminen, 2018). These specific concerns seem fitting, given that anxiety is related to an ambiguous (future) threat. In relation to anxiety disorders, anxiety could potentially be a source of action. Obsessive‐compulsive disorder, which is specified as an anxiety disorder by ICD‐10 (WHO), is clearly action‐oriented. People with this disorder tend to have specific behavioral rituals that aim to relief feelings of discomfort (Soomro, 2012).

This literature review indicates that anxiety influences attitudes, such that it increases a preference for what is known and safe, decreases support for risky policies (Huddy et al., 2007) and that it influences political behavior, such as voting for the safe option (Bäck et al., 2020), leads to increased deliberation (MacKuen et al., 2010), and increases the likelihood of other low‐cost political actions (Denny, 2016). Hence, it seems that anxiety may have some effects both on political attitudes and political behavior, but more research on this link is clearly needed. All in all, there is very little research on how fear, and especially in contrast to anxiety, affects political attitudes and behavior. One reason for this may be that the development of good measures for discrete emotions to be used in self‐report surveys has fallen behind (Harmon‐Jones et al., 2016).

The present study utilizes the Covid‐19 pandemic to explore how different negative emotions, specifically anger, fear, and anxiety, influence political attitudes and action intentions. Specifically, we expect that highlighting certain aspects of the pandemic will evoke specific emotions that in turn will influence policy attitudes and action intentions. Before specifying our hypotheses, we briefly describe the Covid‐19 context and focus particularly on Sweden where the present research was conducted.

## 3 EXPECTATIONS ABOUT EMOTIONS AND POLICIES IN RELATION TO THE COVID‐19 PANDEMIC

### 3.1 Policies in relation to Covid‐19 and the case of Sweden

In an overview of policies implemented in reaction to Covid‐19, Cheng et al. (2020) present data of over 10 000 policy announcements in over 190 countries. The most commonly implemented policy was to close national borders. The second most common policy was to close schools and nonessential businesses. In most cases, the implementations were also compulsory. Such measures undoubtedly affect citizens both individually and as a nation (e.g., Arora & Grey, 2020).

The Swedish approach initially deviated from most other countries since Sweden did not go for a lockdown strategy. The Swedish goal was still to “flatten the curve,” that is, to allow the health care system to keep up and avoid a collapse. Hence, while the means to reach the goal differed, the goal itself was the same across all countries. Whereas most other countries opted for restrictions regulated by law enforcement, the Public Health Agency of Sweden provided recommendations for individual behavior. The term recommendation is heavily culturally dependent and also varies with sender within Sweden. As Prime Minister Stefan Löfven stated in a rare speech to the public on March 22, when the Public Health Agency gives a recommendation, it is expected that the public complies—it is not a choice to do it or not, you should do it. Still, there was no punishment for citizens that did not follow the recommendations, which were, among others, to keep distance, avoid nonessential travels, increase hand hygiene, and to stay at home and work from home as much as possible. Shops and businesses remained open during the initial crisis, as did pre‐schools and basic schools. Later on, more restrictions were enforced and in March 2021, a new “Pandemic law” was implemented giving the Government legal jurisdiction to close shops, gyms, restaurants, etc. This actually means that the Swedish restrictions later on in the pandemic became more similar to the other Western European countries' lockdowns. The relatively lenient initial Swedish approach made Sweden an excellent case for exploring health behaviors during the early stages of the pandemic.

The Covid‐19 pandemic brought with it two main problems that any government had to deal with. First, the acute problem was to restrict the spread of the virus, but a secondary problem to the policies implemented to reach the first goal, was the sudden halt on the economy. Hence, measures to deal with these problems are to some extent contradictory. The question of interest here is when and why people support different kinds of policies speaking to the different problems? We here expect that support for the different kinds of policies should be dependent on the individual's emotional state, which in turn may be evoked by highlighting different aspects of the pandemic. That is, to what extent do emotions mediate the effect of different threats posed by the pandemic, on policy support and political actions?

### 3.2 Hypotheses about emotions and policy support

The most urgent policies that were been discussed in relation to the Covid‐19 pandemic were (a) policies that aimed to restrict the spread of the virus and (b) policies to support the economy. We expect that information focusing on the related threats, spread of the virus and economic collapse, will influence what kind of policies people support, but that the mediating mechanism is via what emotions the threats trigger. The stimuli material focused on either transgressors of the Public Health Agency's recommendations or the spread and danger of the virus.

First, we expect that focusing on transgressors of the recommendations will evoke anger, and that that anger will increase support for policies directed at limiting the spread of the virus, that is, more restrictive policies. This could be seen as a way to “get back” at the transgressors. Anger has been shown to be related to more aggressive policies (Sadler et al., 2005; Skitka et al., 2006), and the policies directed to limiting spread severely limits individual freedom. Hence, we expect that: *focusing on transgressors of the Public Health Agency's* *recommendations should lead to anger, which in turn is likely to increase support for restrictive policies* *(H1a)*. We do not expect that anger will affect support for economic policies.

Second, we expect that focusing on the spread and danger of the virus will evoke fear, which in turn will increase policies that target spread control (restrictive policies). Fear in this case should mainly be related to a fear for the own health. Hence, both anger and rear are predicted to increase restrictive policy support, but for different reasons. We hypothesize that: *focusing on the virus and the strained health care situation should lead to fear, which in turn is likely to increase support for restrictive policies (H1b)*. We do not expect that fear will affect support for economic policies.

Third, we expect that a focus on the spread and danger of the virus will also evoke anxiety, which in turn will increase economic policy support. Given that anxiety should be related to a prolonged concern, compared to fear, we expect that anxiety will have a stronger impact on support for policies related to the economy. The economic threat of the Covid‐19 pandemic is less clear and it is uncertain how life will pan out in the post‐crisis period. Here, we also draw on Fetzer et al. (2020), who found that focusing on the mortality rate of Covid‐19 increased economic concerns in a US population. Hence, we hypothesize that: *focusing on the virus and strained health care situation should lead to anxiety, which in turn is likely to increase support for economic policies (H1c)*.

### 3.3 Hypotheses about emotions and political actions

Anger is an action‐oriented emotion and has long been considered a predictor of political activity, hence we here expect that anger will increase intentions to engage in political actions. We thus hypothesize that: *focusing on transgressors of the Public Health Agency's* *recommendations should lead to anger, which in turn is likely to increase political action intentions* *(H2a)*.

Regarding fear, we expect that fear is not linked to political engagement or possibly even negatively related to engagement, since fear is generally related to avoidance. Because of the exploratory focus or this study, we do not specify a hypothesis for fear and political action intentions.

Since there is very little prior research on anxiety and political engagement, it is not straightforward to hypothesize about its effects. Nonetheless, since anxiety is related to an ambiguous threat and a prolonged emotional state, we believe that anxiety could also be related to increased political action tendencies. However, we make this claim with the precondition that the political activities that we measure here are of a fairly specific nature. Since participating in demonstrations is not an option in relation to the Covid‐19 pandemic, the actions are more in the form of online participation. This would be in line with the study by Denny (2016), who found that financial anxiety was related to political participation when participation was measured as signing an online petition. We tentatively expect that: *focusing on the virus and strained health care situation should lead to anxiety, which in turn is likely to increase political action intentions (H2b)*.

## 4 METHODS AND DATA

### 4.1 Overview of the experimental study

This experimental study designed to evaluate the hypotheses presented above employs two treatments designed to elicit different emotional responses—either anger, or fear and anxiety. The two experimental conditions consisted of short fictive news articles (about 300 words each).

The first article focused on the virus, the limits of the health care system when dealing with too many intensive care patients, the dangers of the virus and estimates of deaths and severe cases. To make it more relevant to the individual, potential consequences of worst case scenarios such that it would entail that almost everybody would be severely affected by the disease—either themselves or someone close to them, were highlighted. Other highlights included that the need for intensive care was not restricted to the elderly population. The aim of this text was to present both concrete and specific stimulus that would elicit fear, where specific measures could help to avoid it, and a more ambiguous stimulus that could elicit anxiety. Even though the virus itself constitutes a specific source of fear, the future consequences of behavioral restrictions are not clear and hence could function to elicit anxiety. We here draw on Fetzer and colleagues (2020), who found that economic anxiety increased following the entrance of Covid‐19 in the US as measured by a substantial increase in google searches for economic recession, as well as survey data. Further, they also found that focusing on the high mortality rate of Covid‐19 led to increased economic worries.

The second article described people who defied authority recommendations to stay at home, and because of this, contributed to unnecessary spread of the virus. The data was collected just before Easter, when the basic school system in Sweden has a one‐week break, which is among certain groups of people often spent going skiing or on holidays abroad. On the 15 March—three weeks before the Easter break—the Ministry for Foreign Affairs issued a recommendation to avoid all nonessential international travels. This led to a fear that people would instead travel within Sweden and hence spread the virus. Especially, focus was on the Stockholm region which was the epicenter of the disease in Sweden, while most other parts of Sweden remained fairly unaffected at this point. Health care workers from around Sweden expressed their concerns that travelers would bring the virus there, and with a limited capacity of health care institutions in smaller regions, they were dreading a disaster. Hence, the text focused on the possibility that some people might disregard the recommendations to stay at home and go traveling to the Swedish skiing resorts, the south of Sweden or the archipelagos regardless of the recommendations. This ensured that the text had a clear focus on specific transgressors where the idea was that their behavior would be perceived as unjust and egoistic and thus elicit anger. Research shows that when violations of justice are severe and transgressors are clearly identified, anger is the prime emotion (Aquino et al., 2001; Bennett & Earwaker, 1994; Bradfield & Aquino, 1999; Darby & Schlenker, 1982; Lickel et al., 2006).

Importantly, everything in the texts were taken from real life news and debates so the texts did not present anything untrue or something that the participants would not encounter just reading a newspaper or watching the news on television. The articles simply summarized different aspects of the pandemic and presented them to the participants. Translations of the texts are found in the Appendix.

We first pre‐tested the material to make sure the conditions did in fact elicit the expected emotions. The pre‐test led to minor changes and also some changes in the emotion scale that we used so that we would be better equipped to capture the emotions of interest. The changes that we made to the stimulus material were small and mainly done to increase emotional reactions. For instance, we made it more explicit that the virus was very dangerous and that most people would probably be affected by it.

### 4.2 Survey measures

Data was collected online by the survey company *Enkätfabriken*. Participants (*N* = 1,072) were randomly assigned one of the two conditions (see more information on the participants below). Data was collected between April 2 and 7, 2020. The study began by information the participants about the study and its purpose, data handling and right to withdraw at any time. Before starting the survey, the participant was required to provide informed consent.

After this, participants responded to demographic questions regarding their age, gender, education, self‐positioning on a left‐right scale *(1 = Clearly to the left, 10 = Clearly to the right),* and a liberal‐conservative scale *(1 = Clearly liberal, 10 = Clearly conservative),* and political interest *(1 = Not at all interested, 10 = Very interested)*. These were used as control variables in our analyses.

The participants then read one of the short news pieces that constituted our main independent variable, and was designed to elicit either anger or fear and anxiety. Following the news piece, we assessed the participant's emotional state. The measure that we used is based on Harmon‐Jones and colleagues' Discrete Emotions Questionnaire (2016). We first pre‐tested the translation of the entire Discrete Emotions Questionnaire in relation to our stimulus material. This pre‐test showed that some items did not translate very well, so we made some changes. For example, the item “Grossed out” was extremely skewed (2.14). Since the disgust scale was not our main interest, we removed this item. When running reliability analyses on the Sadness subscale, the item “Lonely” decreased Cronbach's alpha. Since sadness was not the main interest of our study, we dropped Lonely in the main study.

We also removed some other items from the emotions scale that were of less interest to the present study, to shorten the questionnaire. For instance, we removed the happiness subscale entirely, since it made little sense to rate happiness in relation to Covid‐19. We also removed the relaxation and desire subscales. Finally, we added some items to better capture the emotions we were primarily interested in: anger, fear and anxiety. The question read: The situation with Covid‐19 can give rise to a range of different emotions. In relation to Covid‐19, what emotions do you experience? Then participants rated on scales from *1* *= Do not experience at all* to *7 = Strongly experience*, different emotions. We were mainly interested in the three emotions anger, anxiety and fear but included some other emotion items that potentially could affect the results as well.

Anger was measured with the items rage, anger, irritation and upsettedness. Fear was measured with the items fear, scare, terror and alarm. Anxiety was measured with the items anxiety, nervousness, unpleasantness, worry, helplessness, apprehension and powerlessness. Two items related to anger and fear were removed due to being highly skewed (fury: 1.46, and panic: 1.42). None of the other focal emotion items had a skewness statistic >1, nor outliers >3 standard deviations (see the Appendix for individual skewness statistics). The items were collapsed into three indices and Cronbach's alpha was high in all of them: anger = 0.87, fear = 0.88, and anxiety = 0.90. The other items we included measured sadness: sorrow, sadness, and emptiness (alpha = .74), disgust: disgust and sickness (*r* = .46, *p* < .001), and finally, empathy: empathy and compassion (*r* = .70, *p* < .001).

After the emotion measure, we asked a set of policy opinion questions relating to the economy and limiting the spread (“flatten the curve”) (the order of the two sets of policy items was randomized), and intentions to engage in political actions.

The question regarding policy opinions about the economy read: Sometimes the need to restrain the spread of the virus is contrasted to the risk that the economy is affected. The government has already taken some measures to facilitate for some industries and individuals that are severely affected by the corona crisis. The policy suggestions below have been discussed as additional measures. What is your opinion about them? The scale ranged from *1* *= Very bad suggestion* to *7 = Very good suggestion*. The items were: Economic support to affected companies, Increase sick leave without written medical certificate to one month (a previous measure was to allow sick leave for two weeks without medical certificate, which is normally one week to be eligible for sick leave compensation), Pay employers' part of the sick leave costs, Extra benefits for housing to individuals that have lost their jobs, Abolished demands for housing payments for three months (this measure was actually later implemented until 2022), Lowered sales taxes to stimulate consumption. The items were combined to a mean index, Cronbach's alpha was .77.

The question regarding policy opinions about limiting the spread of the virus read: Below are some suggestions of measures that have been taken in other countries and that are discussed in Sweden to limit the spread of contamination. How good do you think these suggestions are? Answers were again made on 7‐point scales from *1* *= Very bad suggestion* to *7 = Very good suggestion*. The items were: Close pre‐schools and basic schools (the equivalent to high schools and higher education institutions were already switched to distance teaching), Limit the number of people that are allowed to meet to two, Use police and military to ensure citizens follow the restrictions, Forbid nonnecessary travelling within Sweden, Close all restaurants and cafés, Only allow online shopping of groceries, Stop gatherings of more than 10 people, Close the borders for both in‐ and out‐bound travels, Total lockdown that is, people are not allowed to leave the home more than one person at the time for essential errands. The items were combined to a mean index and Cronbach's alpha was .87.

Intentions to engage in political actions were measured with the question: Sometimes people want to affect politics by engaging. In relation to the outbreak of Covid‐19, are there any of the following activities that you could consider doing? The answers were made on a 7‐point scale from *1* *= No, cannot consider doing*, to *7 = Yes, can absolutely consider doing*. The items were: Share information on social media (such as news articles), Post or comment on social media, Sign an online petition, Contact politicians or authorities, Discuss the government and public health agency's measures with friends. The items were combined to a mean index, and alpha was .78.

### 4.3 Participants

Out of the 1,072 participants, 547 (51%) were in the fear/anxiety condition and 525 (49%) in the anger condition. There were 496 men and 505 women in total (71 did not answer the gender question). In the fear/anxiety condition there were 244 men and 271 women, and in the anger condition there were 252 men and 234 women. Mean age was 50 years old, *SD* = 15.57, range 18–88. Age did not differ between conditions, *t*(984) = 0.30, *p* = .76. The sample was fairly highly educated: 1 person had not completed basic schooling, 68 had only basic schooling, 336 had the equivalent of high school, 112 had post high‐school vocational training, 482 had higher education (college or university), 13 had post‐graduate education, and 60 participants did not answer the education question. The distribution of education between the conditions were as following where the fear/anxiety condition is presented first, then the anger condition: No basic schooling: 0/1, Basic schooling: 37/31, high school: 169/167, post high‐school vocational training: 57/55, higher education: 248/234, post‐graduate education: 10/3.

The participants were fairly centrist when it comes to left‐right position, *M* = 5.41, *SD* = 2.32, and liberal‐conservative, *M* = 4.74, *SD* = 2.00. They were also fairly interested in politics, *M* = 5.68, *SD* = 2.54 on the 10‐point interest in politics scale. To make sure that there was no difference in the control variables across the experimental conditions, we *t*‐tested the ideological positioning scales (left‐right, liberal‐conservative) and interest in politics between the conditions. There were no significant differences, *t*s < 1.34, *p*s > .18.

### 4.4 Analytical strategy

Because our theoretical argument is that the experimental conditions lead to different emotional reactions, which in turn affect policy support and political action intentions, we ran mediation models. To test all mediators (all emotions) simultaneously, we ran parallel mediation analysis. This means that both of the experimental conditions were allowed to influence all emotions, and all emotions were allowed to influence the outcome variables. All *p*‐values < .05 were considered significant. We used Hayes' PROCESS macro for SPSS, model 4 to run the analyses (Hayes, 2013, 2018). Before presenting the results from the mediation analyses, we present descriptive results.

## 5 EMPIRICAL RESULTS

### 5.1 Descriptive results

Table 1 shows means and standard deviations on the mediation variables split on condition along with results from separate *t*‐tests.

**TABLE 1:** Means and standard deviations for the emotion indices split on condition

Outcome	Condition		*p*	Cohens' *d*
	Fear/Anxiety	Anger		
Fear	3.23 (1.50)	2.86 (1.40)	<.001	0.26
Anger	2.78 (1.46)	3.00 (1.48)	.016	0.15
Anxiety	3.72 (1.44)	3.39 (1.37)	<.001	0.24
Sadness	3.47 (1.47)	3.49 (1.40)	.03	0.01
Disgust	2.07 (1.23)	2.07 (1.25)	.99	
Empathy	5.22 (1.30)	5.16 (1.34)	.52	

There were significant differences between the conditions on all three of the focal emotion indices. As expected, fear was higher in the fear/anxiety condition focusing on contamination and spread of the virus compared to the anger condition, and anger was higher in the anger condition compared to the fear/anxiety condition. Anxiety was also higher in the fear/anxiety condition compared to the anger condition. Hence, the information in the stimulus texts successfully affected the participants' emotional state. Even though we did not have any expectations about sadness, sadness was significantly higher in the fear/anxiety condition compared to the anger condition. Disgust and empathy did not vary with condition. Hence, we include sadness, but not disgust or empathy, in our main analyses (see the Appendix for analyses including all emotion indices).

Table 2 shows means and standard deviations for the dependent variables, split on condition. There were no significant differences between the conditions on any of the outcome variables, *t*s absolute values <1.5, *p*s > .12.

**TABLE 2:** Means and standard deviations for the outcome variables split on condition

	Condition		Total
	Fear/anxiety	Anger	
Economy policy support	5.41 (1.01)	5.49 (1.05)	5.43 (1.03)
Restrictive policy support	3.60 (1.33)	3.57 (1.29)	3.59 (1.31)
Political actions	3.44 (1.49)	3.58 (1.44)	3.51 (1.47)

Table 3 shows correlations between the emotions and the outcome variables. As can be seen, there were fairly strong correlations between most emotions and opinions and action intentions. All emotions were positively related to policy support for both economic and spread control policies. All emotions were also positively related to political action intentions.

**TABLE 3:** Bivariate correlations between the emotion indices and the outcome variables

	Fear	Anger	Anxiety	Sadness	Disgust	Empathy
Economy	0.14***	0.09**	0.17***	0.14***	0.04	0.26***
Restrictive	0.35***	0.26***	0.29***	0.22***	0.25***	0.07*
Political actions	0.18***	0.26***	0.24***	0.18***	0.14***	0.20***

*** *p* < .001

** *p* < .01

* *p* < .05.

### 5.2 Main analyses—Mediation models

**FIGURE 1:**
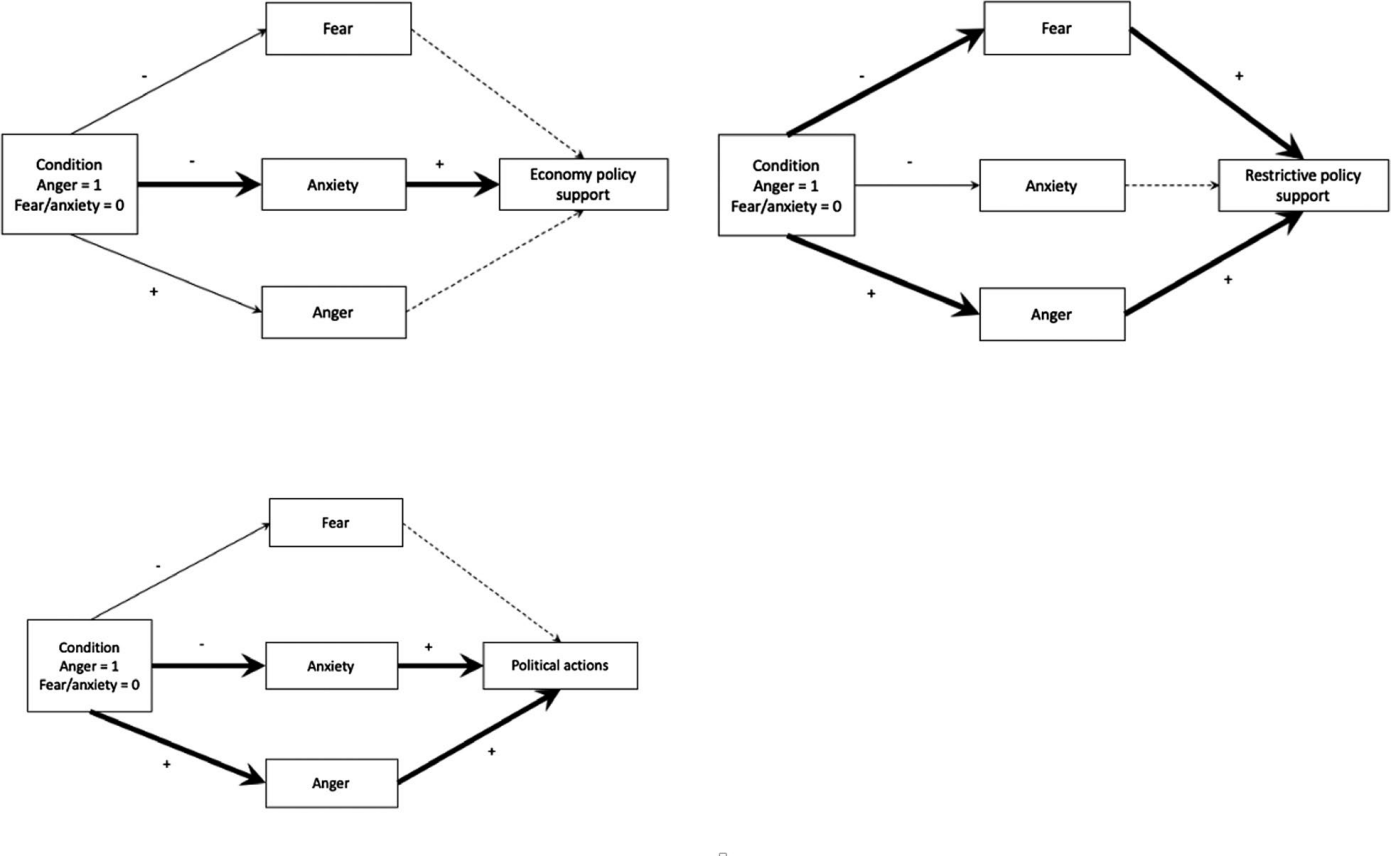
The effect of condition on support for economic and restrictive policies (panel a and b), and political action intentions (panel c), via fear, anxiety, and anger

To test our hypotheses that highlighting different aspects of the Covid‐19 outbreak will lead to different emotional reactions, which in turn affect policy support, and political action intentions, we performed a series of parallel mediation models, one for each dependent variable. In all models, we controlled for age, gender, education, ideological position (both left‐right and liberal‐conservative), and political interest. We also included sadness in all models since sadness was elevated in the fear/anxiety condition. See the Appendix for models with all emotions. The results can be seen in Table 4, which shows the regression results, and Table 5, which shows the bootstrapping results. The effects are illustrated in Figure 1, where panel A and B show the effects on policy support, while panel C shows the effect on Political Actions.

**TABLE 4:** Parallel mediation models for policy support and political action intentions, with emotions as mediators and condition as independent variable

	Anger (M1)	Fear (M2)	Anxiety (M3)	Sadness (M4)	Restrictive policy support (*Y*)	Economy policy support (Y)	Political action intentions (*Y*)
Antecedent	Coeff	Coeff	Coeff	Coeff.	Coeff.	Coeff	Coeff.
Constant	2.66 (0.29)***	2.84 (0.29)***	3.46 (0.28)***	3.29 (0.30)***	2.96 (0.27)***	5.25 (0.23)***	2.25 (0.30)***
Condition	0.16 (0.05)***	−0.15 (0.05)***	−0.13 (0.05)**	−0.09 (0.05)	0.03 (0.04)	0.05 (0.03)	0.08 (0.04)
Anger (M1)	–	–	–	–	0.08 (0.03)*	0.00 (0.03)	0.13 (0.04)***
Fear (M2)	–	–	–	–	0.27 (0.05)***	−0.03 (0.04)	−0.07 (0.06)
Anxiety (M3)	–	–	–	–	−0.03 (0.06)	0.13 (0.05)**	0.24 (0.07)***
Sadness (M4)	–	–	–	–	0.01 (0.04)	0.02 (0.03)	−0.00 (0.05)
Gender (C1)	0.40 (0.10)***	0.59 (0.10)***	0.61 (0.09)***	0.48 (0.10)***	−0.01(0.08)	0.13 (0.07)	0.10 (0.09)
Age (C2)	−0.01 (0.00)***	−0.00 (0.00)	−0.00 (0.00)*	0.00 (0.00)	−0.00 (0.00)	0.00 (0.00)	−0.01 (0.00)***
Education (C3)	−0.09 (0.05)*	−0.05 (0.05)	−0.04 (0.04)	−0.03 (0.04)	−0.13 (0.04)***	−0.11 (0.03)***	0.05 (0.04)
Left‐right (C4)	0.02 (0.02)	−0.03 (0.02)	−0.04 (0.02)	−0.02 (0.02)	−0.02 (0.02)	0.01 (0.02)	−0.02 (0.02)
Liberal‐Conservative (C5)	0.11 (0.03)***	0.10 (0.03)***	0.07 (0.03)**	0.05 (0.03)	0.11 (0.02)***	−0.02 (0.02)	−0.07 (0.02)*
Political interest (C6)	0.03 (0.02)	0.03 (0.02)	0.04 (0.02)*	0.03 (0.02)	−0.05 (0.02)*	−0.02 (0.01)	0.16 (0.02)***
	*R*^2^ = .08	*R*^2^ = .07	*R*^2^ = .07	*R*^2^ = .04	*R*^2^ = .18	*R*^2^ = .06	*R*^2^ = .18
	*F*(7,943) = 11.21***	*F*(7,943) = 9.45***	*F*(7,943) = 9.41***	*F*(7,943) = 4.90***	*F*(11,939) = 18.52***	*F*(11,939) = 5.27***	*F*(11,939) = 18.73***

Note

Condition is dummy coded with fear/anxiety = 0, anger = 1.

Gender is dummy coded with men = 0, women = 1.

*** *p* < .001

** *p* < .01

* *p* < .05.

**TABLE 5:** Direct and indirect effects of condition on policy support and political action intentions from bootstrapping with confidence intervals in parenthesis

	Restrictive policy support	Economy policy support	Political action intentions
Direct effects	0.02 (−0.05; 0.10)	0.05 (−0.02; 0.11)	0.11 (−0.06; 0.29)
Indirect effects			
Fear	−0.04 (−0.07; −0.01)	0.00 (−0.04; 0.00)	0.03 (−0.01; 0.08)
Anger	0.01 (0.001; 0.03)	−0.00 (−0.01; 0.01)	0.04 (0.01; 0.08)
Anxiety	0.00 (−0.02; 0.02)	−0.03 (−0.07; −0.002)	−0.05 (−0.12; −0.01)
Sadness	0.00 (−0.01; 0.01)	0.01 (−0.01; 0.02)	−0.00 (−0.03; 0.02)

Note

Level of confidence for all confidence intervals is 95%. Results are based on 5,000 bootstrap samples.

As can be seen there is an effect of condition on all three focal emotions. Anger is higher in the anger condition, and fear and anxiety are higher in the fear/anxiety condition. In these models, the effect of condition on sadness did not quite reach conventional significance levels (*p* = .052).

Gender had a significant effect on all emotions such that women reported stronger emotional experiences overall. There was a weak negative effect of age on anger and anxiety indicating that both anger and anxiety tended to decrease with higher age. Higher education was related to less anger but not to any other emotion. Ideological position on a left‐right scale had no effect on emotions. However, people who were more conservative, reported stronger emotional experiences overall, except for sadness. Higher political interest was weakly related to anxiety.

Hypothesis H1a stated that when information focused on people who disobeyed the Public Health Agency's recommendation, people would experience anger and, in turn, anger would predict support for more restrictive policies. As can be seen in Table 4, the coefficient for anger on support for restrictive policies was significant, in support of H1a. The results indicate that the condition influences the outcome variable via anger.

Hypothesis H1b stated that when information focused on the virus and the strained health care situation, this would elicit fear, which in turn would predict support for restrictive policies. Again, the coefficient between fear and restrictive policy support was significant, indicating support for H1b.

Hence, both anger and fear were related to increased support for more restrictive policies, so called “flatten the curve”‐policies. Age, gender and left‐right position was unrelated to support for restrictive policies, while higher education and higher political interest was related to lower support, more conservative self‐positioning was related to more support.

Hypothesis H1c stated that information focusing on the virus and health care situation should also elicit anxiety, and this in turn should be related to support for economic policies since anxiety is rather related to an ambiguous stimulus. As can be seen, the coefficient for anxiety on economy policy support was significant, such that higher anxiety was related to more support for economy policies. Again, the condition influenced policy support via anxiety, supporting hypothesis H1c.

There were no effects of the other emotions on economy policy support. Again, education was negatively related to policy support such that higher education was related to lower support for economy policies. None of the other control variables exerted a significant effect.

Finally, we expected the focal emotions to have different effects on political action intentions. H2a stated that information focused on transgressors would lead to anger, and anger would increase political action intentions. As can be seen in the last column of Table 4, there was a positive effect of anger on political action intentions indicating that angry people were more likely to be willing to engage politically. H2b stated that information about the virus leading to anxiety should also increase participation intentions. In line with this, we found that the coefficient for anxiety on political action intentions was positive, indicating that anxious people were more willing to engage politically. Neither fear nor sadness was significantly related to political action intentions.

Interestingly, there were weak or no effects of ideological position on the outcome variables. Left‐right position had no effect on either policy or political action intentions. Liberal‐conservative position had a positive effect on restrictive policies such that individuals who rated themselves as more conservative were more in favor of restrictive policies. These results are in line with research on conservatism and authoritarianism and restrictive policies in general (McKeever, 2020; Poteat & Mereish, 2012). There was also a weak negative effect of conservatism on political actions, which is also in line with previous research on political engagement and ideology. However, these results also indicate that the policies related to the handling of the Covid‐19 pandemic do not follow partisan lines. Not even the traditionally economic left‐right scale had any influence on the economic policies, which largely follow from ideological differences. These results are in line with some previous research experimentally testing partisan endorsement of Covid‐19 policies in the US, where Gadarian et al. (2020) did not find any partisan effects on health behaviors or policy attitudes.

Finally, there was a weak negative effect of political interest on restrictive policy support such that those who were more interested in politics were less supportive of restrictive policies, and a positive effect of political interest on political action intentions such that increased political interest was also related to increased intentions to engage. This latter result is in line with results found in previous research on political interest and engagement, both offline and online (Gibson & Cantijoch, 2013; Oser et al., 2013).

## 6 GENERAL DISCUSSION

The present research shows that different threatening aspects of the Covid‐19 pandemic evoke different emotional reactions, which in turn affect support for different policies and intentions to engage in political actions. The experiment consisted of two conditions, which highlighted either transgressors of the Public Health Agency's recommendations, and was designed to elicit anger, or the danger of the virus, the strained health care situation, and uncertainty of deaths and severely ill citizens, designed to elicit fear and anxiety.

Using an adapted variation of Harmon‐Jones et al.'s (2016) measurement of discrete emotions we found that the information provided to participants elicited the expected emotional reactions. Subsequent modeling showed that both fear and anger led to support for more restrictive policies, but for arguably different reasons. In the anger condition, the information was focused on justice violations by specific individuals which led people to become angry. Restrictive policies for these people should be related to restriction of transgressors' possibility to violate recommendations—to restore justice and fairness. For fearful individuals, however, restrictive policies should be related to a desire to limit the spread of the disease to decrease the risk to the own health. Participants who reported higher levels of fear were informed about the danger of the virus and the possibility of overwhelming the health care system. For these individuals, restrictions should mainly restore a sense of control and security.

Relatedly, a recent paper (Harper et al., 2020) showed that fear was related to increased public health behaviors such as social distancing and increased hand hygiene. Even though previous research has shown that fear may lead to support for less aggressive policies (Lerner et al., 2003), which could be considered contradictive to our results where fear was related to support for restriction of individual freedom, we argue that these results are not incompatible. First, the aggressive policies studied in earlier research are related to intergroup threat (i.e., 9/11 terrorist attacks), while the current situation has no social group as enemy, but a virus. Thus, restrictions of individual freedom should not be conceived of as an aggressive policy, but rather a precautionary measure, which also has been shown to increase when individuals experience fear (Brader & Marcus, 2013; Lerner et al., 2003). Moreover, fear was not assessed in the earlier study, making comparisons across studies difficult since the participants may have been driven by other emotions.

Even though fear and anger are fundamentally different emotions, they are both high‐arousal emotions (Marcus et al., 2000). This could explain why they both are related to restrictive policies, which focus on the situation here and now, but not economic policies. To support economic policies, individuals need to extend their focus beyond the immediate situation and evaluate what might come after the pandemic has passed. Such an extended focus may be difficult under states of high‐arousal, which tends to narrow the attentional span (Brader & Marcus, 2013). Anxiety, however, is related to increased attention to information and less reliance on prior convictions (Brader & Marcus, 2013). Similarly, while anger strengthens reliance on prior convictions, anxiety in fact had the opposite effect, undermining such use of heuristics (Banks & Valentino, 2012).

In line with previous research on emotions and political activity (see, e.g., Brader & Marcus, 2013), we found that anger increased intentions to engage in political actions. For instance, Brader et al. (2010) found that when faced with a potentially deadly viral outbreak, angry citizens were more likely to engage politically by for instance contacting officials, and demanding investigations and prosecution of those responsible for the outbreak. Fearful individuals, however, were more likely to engage in protective behaviors such as wearing a face mask, increasing hand hygiene and getting informed.

Interestingly, we also found that intentions to engage politically was mediated by anxiety. The political psychology research to date has not focused particularly on anxiety (Brader & Marcus, 2013), although some exceptions exist. For instance, Denny (2016) found that financial anxiety led to increased participation in the form of signing an online petition. Similarly, in the present study, the participation items mainly concerned actions that could be performed online, such as sharing material, signing petitions and contacting authorities. Another reason to assume that anxiety may increase participation tendencies is that anxious people may tend to see beyond the immediate situation, and research show that anxiety heightens attention to information. Even though previous research also show that fear is related to precautionary planning (see, e.g., Brader & Marcus, 2013; Lerner et al., 2003), it may be confounded with anxiety since these emotions rarely have been separated in previous research.

Compared to fear, which is focused on seeking out immediate security from the present situation, anxiety should relate more to future prospects. For instance, in the APA diagnostic manual, DSM‐5, anxiety is described as a concern about future events. This idea is further corroborated in our study, where anxiety was related to economic policy support rather than restrictive policy support. Together, this indicates that anxious people have a different focus than fearful people, or angry for that matter.

Relating these results to trait anxiety, or anxiety disorders, they make intuitive sense. Highly anxious people tend to ruminate over certain aspects and hence seek out information and plan ahead. Such behavior should be facilitated in a state of anxiousness compared to fear, where the response is to seek out immediate protection from the fear‐arousing stimulus. Anxiety can also be relieved by performing certain actions. For instance, clinical anxiety syndromes, such as obsessive‐compulsive disorder, often manifest as rituals, where the goal of certain actions is to decrease anxiety (such as excessive hand washing). This could also explain why anxious people scored higher on intentions to engage politically—such engagement could be a way to reduce the unpleasant state of anxiety.

An important result of the present research is that we did not find any main effects of condition on the outcome variables. This is important because much previous research in political psychology have not assessed emotions as mediators, which could explain previous conflicting results, or null results. In a recent pre‐print, for example, Gadarian et al. (2020), did not find any effects of highlighting different aspects of Covid‐19 on policy suggestions in a US setting. Lambert and colleagues (2019) refer to “oppositional mediation” (Kenny, 2017), when discussing that a specific stimulus may elicit different positively correlated emotions, such as anger and sadness, which in turn may have opposite effects on a specific outcome variable, such as a certain policy. This would lead to null effects if emotions are not considered in the model.

This study fills an important gap in the literature on emotions and political attitudes and behavior, and shows that the same imminent threat (Covid‐19) could lead to widely different emotional reactions among individuals depending on framing, which in turn lead to different, or even the same outcomes, but for different reasons. This study also highlights the importance of emotions in political life, and that researchers in political psychology should increasingly pay attention to individuals' emotional states. In addition, it is fruitful to separate between different negative emotions, even beyond splitting up anger and other negative emotions. We have here focused specifically on anger, fear and anxiety, but depending on the topic and research question, other emotions may also be relevant, such as pride or enthusiasm (Brader & Marcus, 2013; Lambert et al., 2019). Fear and anxiety, even though they may elicited by the same information, lead to very different outcomes—both support for different policies and political action intentions. This means that much previous research in the field of political psychology that has used threat to manipulate attitudes and behavior may be confounded with differential effects of emotions that have not been measured.

### 6.1 Limitations and suggestions for future research

Some limitations of the present study should be mentioned. Even though we did our best to create stimulus material that would function to elicit specific emotions, the stimulus material was consistent within each condition. That is, the results would have been stronger and more generalizable should there have been different versions of the texts that elicited fear/anxiety and anger respectively. For instance, it is possible that reactions would have been different if the triggering agent was different—if people were angry at how the Government handled the situation, they might have responded differently to the outcomes.

Another, related issue, is that the texts differed in content. This means that we cannot be certain that there are no other factors that may be responsible for the results, besides the emotional reactions. Because the topic of the two texts were both highly discussed and presented in the media at the time, we cannot know if participants used only the information provided in the texts to form their policy attitudes, or if they also relied on prior knowledge. This too, is an empirical question for future research.

Both fear and anxiety were elicited by the information focusing on the strained health care system. We do not know exactly what information in the text evoked fear and what evoked anxiety, or if some underlying variable, such as individual differences in proneness for fear or anxiety, or prior knowledge, played a role in the different emotional reactions. This could be seen as problematic, but we deemed it a necessary trade‐off to make the stimulus material externally valid. Future research should go more closely into exploring exactly how anxiety and fear can be manipulated, separately. We did not include a control condition, which would be beneficial for providing base‐line emotions, and compare how the experimental conditions differed. Nonetheless, given that the sample was fairly large and that all participants can be assumed to have had access to the same information, the fact that we find significant effects speak for the validity of the stimulus material and its capacity to evoke different emotions.

Also, the differences in emotions between conditions were also fairly small, especially the difference in anger. Upon creating new stimulus material, it would be good to try to better differentiate between emotions evoked by the different conditions. Nevertheless, this study shows that even subtle changes in the focus on the Covid‐19 reporting do influence what emotions citizens experience, which in turn may affect what policies they support.

Finally, it should be noted that there are some inherent problems associated with measuring emotions in the format we did here, that is, when the participant is asked to rate a host of emotions. Because they read all emotions and rate them, this may remind them that they also feel other emotions that they had not initially thought of. Therefore, it would be good to develop a measure of emotions that is less intrusive. As the measure looks now, there needs to be very strong effects between the eliciting stimuli for the scale to fall out on the expected dimensions, which is difficult to achieve when the emotions are close to each other. For instance, it may be difficult for a participant to separate if they feel worried or alarmed, and probably they do experience both to some extent, and as the format is now, participants may overestimate their experience on some, closely related, emotions.

### 6.2 Conclusions

The present research has both theoretical and societal relevance. We contribute to the research on emotions and how they are related to political attitudes and behavior. Specifically, fear and anxiety have different effects on both policy preferences and political action intentions. Societally, the results are important for understanding how citizens respond to different reports of the pandemic, and how the responses may elicit support for different policies and political actions, which has consequences for health related behaviors. The fast‐track emotions fear and anger seem both to increase support for restrictive policies that aim to reduce the spread of the virus and “flatten the curve.” However, since these are high‐arousal emotions, their effect is unlikely to be prolonged. Anxiety, in contrast, probably does have a prolonged effect, but this emotional response was unrelated to restrictive policies.

## CONFLICT OF INTEREST

The authors declare that the research was conducted in the absence of any commercial or financial relationships that could be construed as a potential conflict of interest.

## 1

**TABLE A1:** Skewness statistics for the focal emotion items

Item	Skewness
*Anger items*	
Rage	0.80
Anger	1.00
Irritation	0.35
Upsettedness	0.31
*Fear items*	
Fear	0.26
Scare	0.48
Terror	0.91
Alarm	0.23
*Anxiety items*	
Anxiety	0.84
Nervousness	0.40
Unpleasantness	−0.17
Worry	−0.13
Helplessness	−0.07
Apprehension	0.18
Powerlessness	0.15
*Sadness items*	
Sadness	
Sorrow	0.01
Sadness	−0.15
Emptiness	0.40

**TABLE A2:** Parallel mediation models including all emotions

	Anger (M1)	Fear (M2)	Anxiety (M3)	Sadness (M4)	Disgust (M5)	Empathy (M6)	Restrictive policy support (*Y*)	Economy policy support (*Y*)	Political action intentions (*Y*)
Antecedent	Coeff	Coeff	Coeff	Coeff.	Coeff.	Coeff.	Coeff.	Coeff	Coeff.
Constant	2.82 (0.28)^***^	2.69 (0.28)^***^	3.33 (0.27)^***^	3.20 (0.28)^***^	2.35 (0.24)^***^	4.87 (0.25)^***^	2.96 (0.29)^***^	4.61 (0.24)^***^	1.79 (0.32)^***^
Condition	0.31 (0.09)^***^	−0.30 (0.09)^**^	−0.26 (0.09)^**^	−0.18 (0.09)	0.04 (0.08)	−0.03 (0.08)	0.06 (0.08)	0.08 (0.07)	0.15 (0.09)
Anger (M1)	–	–	–	–	–	–	0.07 (0.05)	0.03 (0.03)	0.15 (0.04)^***^
Fear (M2)	–	–	–	–	–	–	0.27 (0.05)^***^	−0.02 (0.04)	−0.06 (0.06)
Anxiety (M3)	–	–	–	–	–	–	−0.03 (0.06)	0.10 (0.05)^*^	0.22 (0.07)^***^
Sadness (M4)	–	–	–	–	–	–	0.00 (0.04)	−0.03 (0.03)	−0.04 (0.05)
Disgust (M5)	–	–	–	–	–	–	0.04 (0.04)	−0.02 (0.04)	−0.04 (0.05)
Empathy (M6)	–	–	–	–	–	–	0.01 (0.03)	0.19 (0.03)^***^	0.15 (0.04)^***^
Gender (C1)	0.40 (0.10)^***^	0.59 (0.10)^***^	0.61 (0.09)^***^	0.48 (0.10)^***^	0.03 (0.08)	0.50 (0.09)^***^	−0.00 (0.08)	0.07 (0.07)	0.04 (0.09)
Age (C2)	−0.01 (0.00)^***^	−0.00 (0.00)	−0.00 (0.00)^*^	0.00 (0.00)	−0.01 (0.00)^***^	0.00 (0.00)	−0.00 (0.00)	0.00 (0.00)	−0.01 (0.00)^***^
Education (C3)	−0.09 (0.05)^*^	−0.05 (0.05)	−0.04 (0.04)	−0.03 (0.04)	−0.05 (0.04)	−0.00 (0.04)	−0.13 (0.04)^***^	−0.11 (0.03)^***^	0.05 (0.04)
Left‐right (C4)	0.02 (0.02)	−0.03 (0.02)	−0.04 (0.02)	−0.02 (0.02)	0.00 (0.02)	−0.05 (0.02)^*^	−0.02 (0.02)	0.02 (0.02)	−0.01 (0.02)
Liberal‐Conservative (C5)	0.11 (0.03)^***^	0.10 (0.03)^***^	0.07 (0.03)^**^	0.05 (0.03)	0.10 (0.02)^***^	−0.05 (0.02)^*^	0.11 (0.02)^***^	−0.01 (0.02)	−0.06 (0.02)^*^
Political interest (C6)	0.03 (0.02)	0.03 (0.02)	0.04 (0.02)^*^	0.03 (0.02)	0.00 (0.02)	0.07 (0.02)^***^	−0.05 (0.02)^*^	−0.03 (0.01)^*^	0.15 (0.02)^***^
	*R*^2^ = .08	*R*^2^ = .07	*R*^2^ = .07	*R*^2^ = .04	*R*^2^ = .05	*R*^2^ = .07	*R*^2^ = .18	*R*^2^ = .11	*R*^2^ = .19
	*F*(7,943) = 11.21^***^	*F*(7,943) = 9.45^***^	*F*(7,943) = 9.41^***^	*F*(7,943) = 4.90^***^	*F*(7,943) = 7.23^***^	*F*(7,943) = 9.76	*F*(13,937) = 15.72^***^	*F*(13,937) = 8.31^***^	*F*(13,937) = 17.47^***^

*** *p* < .001

** *p* < .01

* *p* < .05.
